# Comparison of Dual Task Training Versus Aerobics Training in Improving Cognition in Healthy Elderly Population

**DOI:** 10.7759/cureus.29027

**Published:** 2022-09-11

**Authors:** Purva H Mundada, Ragini M Dadgal

**Affiliations:** 1 Physiotherapy, Ravi Nair Physiotherapy College, Datta Meghe Institute of Medical Sciences (Deemed to be University), Wardha, IND

**Keywords:** montreal cognitive assessment, trail making test b, trail making test a, aerobic training, dual task training, cognitive function

## Abstract

Background

Cognitive impairments, particularly in old age, are pervasive and occur because of both normal and pathological senescence. Engaging in some routine bodily activities combined with activities that stimulate cognitive skills appears beneficial in increasing cognitive resistance to degenerative processes of the brain. Dual-task training (DTT) by combining motor and cognitive activities causes improvement, particularly in executive function, working memory and divided attention, whereas aerobic exercise training (AET) plays an important role in improving executive function, attention, and memory. In this study, we attempted to compare the efficiency of DTT versus AT in improving cognitive function in healthy older individuals.

Methods

Forty healthy older adults between 60 and 70 years of age who met the inclusion criteria participated in this study. They were randomly split into two groups A and B. Group A (64.05±3.17 years) received DTT three times a week, whereas group B (65.50±3.44 years) received AT five times a week. Both training programs were conducted for six weeks. Cognitive function was assessed using Trail Making Test (TMT)-A, TMT-B, and Montreal Cognitive Assessment (MoCA). The assessment was done at baseline (first day of intervention), on completion of the third week, and again at the end of the training session i.e., the sixth week. The Chi-square test and the student's paired and unpaired t-tests were used for statistical analysis with a level of significance P<0.05.

Discussion and result

Betterment in cognitive functions was evident after six weeks of DTT and AET. Post-intervention improvements were noted in TMT-A, TMT-B, and MoCA scores in both groups A and B (P>0.0001). However, the difference between the pre and post-intervention scores was greater for group A compared to group B indicative of remarkable improvements in cognitive function in group A.

Conclusion

The current study demonstrated that both DTT and AET are notably efficient in improving cognitive function in a healthy elderly population. However, in comparison, DTT was significantly more effective than AET (P<0.05). This shows that six weeks of DTT is effective in improving cognitive function and slowing age-associated cognitive decline in older adults.

## Introduction

India's geriatric population is on the rise [1]. Around 10 crore Indians are more than 60 years of age. The average lifespan of the Indian population has expanded from 32 years in 1947 to 54 years in 1980 and 70.19 years in 2022 [2,3]. As per the World Health Organization (WHO), the global population aged 60 and more is anticipated to increase from 900 million in 2015 to 2000 million by 2050 [4]. Approximately 18 million people worldwide are said to be dealing with age-related mental health concerns. As stated by the WHO, this figure will increase to 34 million by 2025 [2].

Aging-related degenerative processes affect brain structure and function. Cognitive decline is practically ubiquitous among elderly people, and it worsens as they become older [5]. Memory loss, deterioration in executive function (EF), and poorer motoric speed and precision, are some of the cognitive deficits that people would almost certainly encounter as they get older [6]. Cognitive function (CF) is a mental process in humans that includes attention, perception, reasoning, knowledge, and memory [6-9]. Additionally, cognitive processes such as perception, learning, memory, attention, problem-solving activities, and psychomotor functions (reaction time, movement timing, and action speed) can be thought of as phases in information processing [2,6]. For functional independence, cognition is crucial [10]. CF is influenced and affected by many factors throughout a person's life. Reduced CF is linked to a sedentary lifestyle and physical inactivity [11]. According to the frontal lobe hypothesis of aging, there is a considerable decline in EF as people get older. Lack of physical activity and mentally challenging activities are two main modifiable risk factors that cause cognitive decline [5]. Physical activity improves the brain's cognitive reserve capacity, thus reducing the harmful impact of aging as well as the chance of acquiring neurological disorders and dementia. It can, for example, increase the execution capacity and information processing speed [12].

Physical activity has recently reawakened interest in its link to increasing brain capacity and its impact on CF, which inevitably declines with age. As a result, it's critical to concentrate on motor activities requiring attention and combine both physical and cognitive abilities (dual task). This could be a novel marker for detecting physical and cognitive frailty in the elderly [12]. Dual task refers to the execution of two distinct tasks concurrently or in quick order. The central EF system plays a role in the dual task activity. It has been established that combining dual-task training (DTT) with physical activity improves EF, as well as general CF. DTT influences brain activity during cognitive processes because it consists of two concurrently performed tasks, typically requiring distinct forces that must be completed all at once or in fast succession [13]. It counteracts the age-related decline in execution, which is vital in everyday life. Many researchers employ simultaneous DTT to increase multitasking abilities and its consequences on EF by performing various tasks and training [14]. The frontal lobe controls EFs and these are essential in a complicated target-directed attitude. They include processes like suppressing an automatic reaction, switching between numerous activities, upgradation of data in working memory (WM), and synchronizing multiple tasks. The process of aging has been proven to affect EF earlier, and it is identified as a key indicator of retained instrumental activities of daily living (IADL) such as meal preparation, financial management, housework, and medicine administration. As a result, these functions are critical for older persons to be functionally independent. The ability to synchronize different tasks, implying the ability to split attention among several tasks, has piqued a great deal of curiosity in previous centuries, particularly in the elderly. Although attention-sharing capabilities deteriorate as the individual gets older, they are nevertheless necessary for various activities of daily living (ADL). In fact, complex motor skills like walking need divided attention, and deficiencies in the capacity of synchronizing multiple tasks are linked to an increased risk of falling. Enhancing EF, particularly divided attention, is thus a viable strategy to promote functional independence and well-being in the elderly, and should be a key target in intervention trials [15].

When intellectually demanding tasks are paired with multimodal physical exercises, performing a cognitive and motor activity (dual task) at the same time appears particularly beneficial for improving CF in healthy elderly people [5]. In brain plasticity, physical training (PT) and cognitive training (CT) may perform distinct but complementary roles [16]. PT boosts the brain's metabolic activity, but this gain can only be used if there is a need for it. Challenging the brain and its particular cells in the context of a specific cognitive activity generates a such type of demand. It is plausible to believe that multimodal training slows down the senescence of the brain, as evidenced by improved cognitive functioning and postponement in cognitive decline, through this mechanism [17]. In other words, PT improves brain metabolism and plasticity, whereas CT exploits and reinforces the improved metabolism and directs brain plasticity by increasing mental demands. If the processes underlying gains in CF differ in PT and CT, then integrating both of them in one intervention might increase the advantages over a single training mode [16].

Aerobic activities increase physical activity levels by increasing the proficiency of the circulatory system to deliver oxygen and the ability or efficacy with which skeletal muscles use it [18]. Several studies have shown that aerobic exercise, which involves the usage of O2 and the sustained action of large muscle groups, can boost EF, processing speed, attention, and memory in healthy elderly people [19].

According to human research trials, aerobic exercise training (AET) has the biggest influence on spatial memory, WM, and executive attention. Other studies have found that physically active individuals perform better in visual figure recognition, spatial memory, and attentional control (i.e., the Eriksen flanker task) [20]. Increased cardiovascular endurance and circulation in the brain lead to better utilization of oxygen and glucose, increased neurogenesis stimulation, and increased synaptic interconnections [12]. There is a link between physical fitness and cognitive performance in women aged 70-80 years and AET improves verbal and spatial memory as well as verbal-auditory learning [12,21].

Previous research studies related to cognitive aspects of aging have found that as people age, their brain structure and function change, involved in EF and the attention system, as well as WM areas [5,11,12]. As a result, it's vital to find and apply efficient ideas and tactics that might aid in the retention and even enhancement of cognitive abilities in the elderly [13].

## Materials and methods

**Figure 1:**
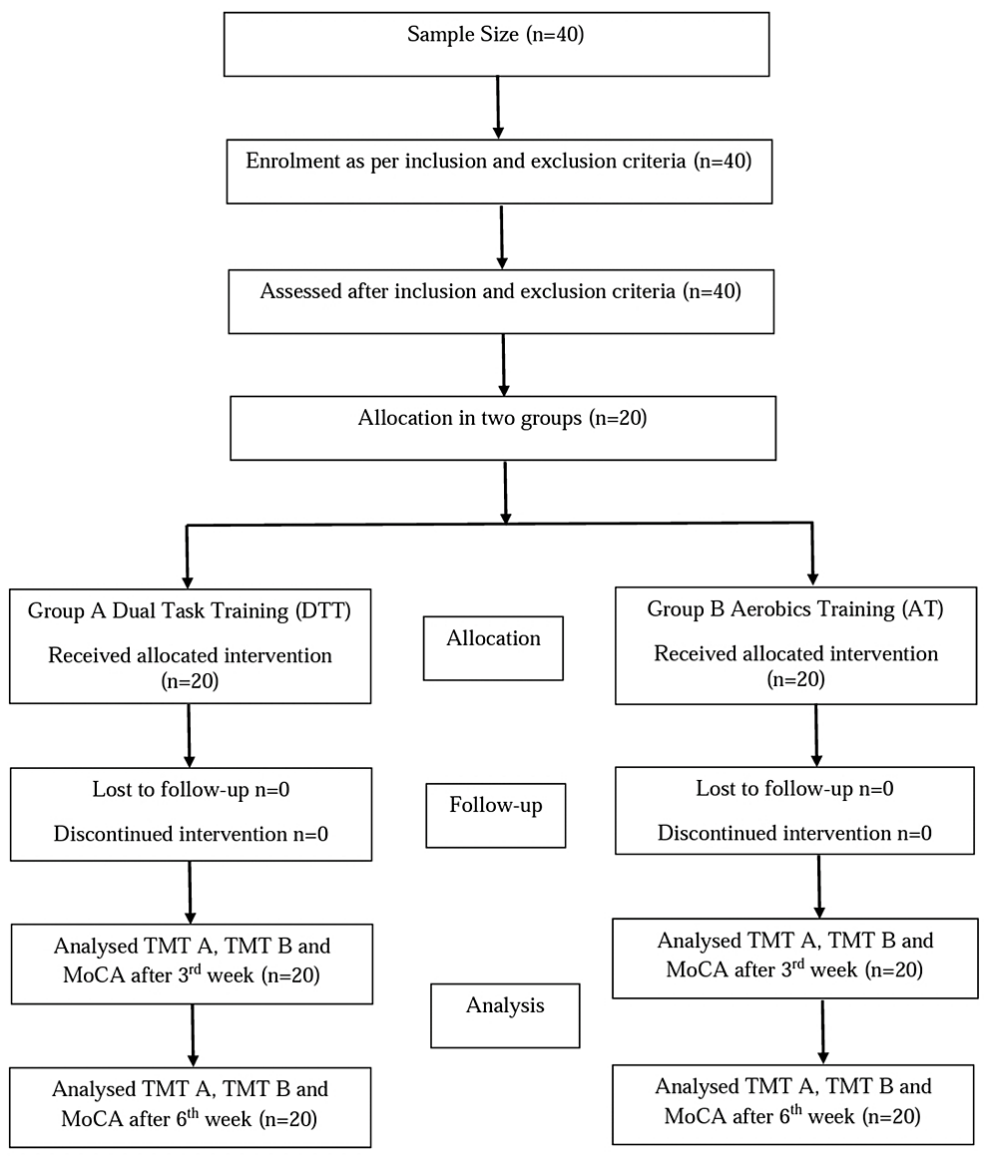
Flowchart of the study procedure TMT-A: Trail Making Test A; TMT-B: Trail Making Test B; MoCA: Montreal Cognitive Assessment

After receiving approval from the Institutional Ethics Committee of Datta Meghe Institute of Medical Sciences (DMIMS) (Deemed to be University), Wardha, India (approval number: DMIMS[DU]/IEC/2021/367), the study was conducted in the neuro-physiotherapy outpatient department (OPD) of Acharya Vinoba Bhave Rural Hospital, Wardha, Maharashtra, India. Informed consent was obtained, arbitrary data was gathered, and a preliminary evaluation was done to determine whether or not the individuals met the inclusion and exclusion criteria. The inclusion criteria were that the participants had to be of either gender and between the ages of 60-70, not taking any medicines known to impair cognitive performance (such as benzodiazepines, antidepressants, or other central nervous system agents) [22], without a known CNS disease, such as thyroid disorders, multiple sclerosis, Parkinson's disease, stroke, severe hypertension (systolic blood pressure greater than 180, diastolic blood pressure greater than 110), or diabetes, six-minute walk distance 592-630 meters for male and 511-558 for female [23], be able to understand and follow instructions, and be willing to participate in the study. People who had any central or peripheral nervous system involvement, severe cognitive impairments, Mini‐Mental State Examination (MMSE) score below 26, musculoskeletal disorders such as rheumatoid arthritis, life expectancy less than three months, severe auditory and visual defects, and those who were currently registered in another clinical trial were all excluded from the study. The selected participants were explained the objectives and methods of the study. Using the sequentially numbered, opaque, sealed envelope (SNOSE) technique, the participants were randomly assigned to either group A or group B by simple random sampling. The primary researcher and a physiotherapy intern conducted the randomization and allocation. The study's enrollment, intervention, and evaluation timeline followed the Standard Protocol Items: Recommendations for Interventional Trials (SPIRIT) 2013 guidelines. A flowchart of the study procedure is depicted in Figure 1.

Outcome measures

A physiotherapy undergraduate student who was aware of the study but blinded to the intervention evaluated the following outcome measures before and after the intervention.

Trail Making Test (TMT) A and B

TMT is a neuropsychological test that combines visual scanning and working memory. TMT is broken into two parts: TMT-A (rote memory or WM) and TMT-B (EF). Each of the two parts consists of 25 circles scattered across a sheet of paper. Part A contains circles numbered from 1-25, and the individual should connect the numbers by drawing lines in ascending order. Part B contains circles with both numbers (1-13) and letters (A-L); the individual must draw lines to connect the circles in ascending order, with the added challenge of alternating between the numbers and letters (for example, 1-A-2-B-3-C, etc.). Individuals must be encouraged to connect the circles as rapidly as feasible. The test results are recorded as the time required for completing the test [24].

Montreal Cognitive Assessment (MoCA)

MoCA is used to assess general CF. It examines cognitive processes such as: “memory,” “language,” “EFs,” “visuospatial abilities,” “calculation,” “abstraction,” “attention,” and “concentration.” It has a total score of 30, with higher numbers indicating better CF. A normal score is regarded to be 26 or higher. A score of 19 to 25 is an indicator of mild cognitive impairment (MCI) [25,26].

Interventions

TMT-A, TMT-B, and MoCA tests were carried out before and after the intervention. Subjects in Group A executed cognitive-motor DTT in which they performed cognitive activities alongside motor ones while subjects in Group B performed AT.

Group A: DTT

In the cognitive-motor DTT, participants performed cognitive activities in conjunction with motor activities. There was evidence of improvement in cognition within four weeks of the intervention [22], whereas another study concluded positive results within six weeks of the intervention [27]. The participants were given 45-minutes training sessions, three times a week for six weeks, taking into account the data given in Table 1.

**Table 1:** Description of the studies including population and duration of intervention that were used as a reference while designing an intervention protocol for the present study

Study	Population	Duration of intervention
Nouchi et.al., 2013 [22]	Healthy older adults (60 years or older)	3 times/week for 4 weeks
Castells-Sánchez et.al., 2020 [19]	Healthy physically inactive older adults (50-70 years old)	45 min, 5 times/week for 12 weeks
Taşvuran Horata et.al., 2020 [27]	Community-dwelling older individuals (60-75 years old)	60 min, 2 times/week for 6 weeks

**Figure 2:**
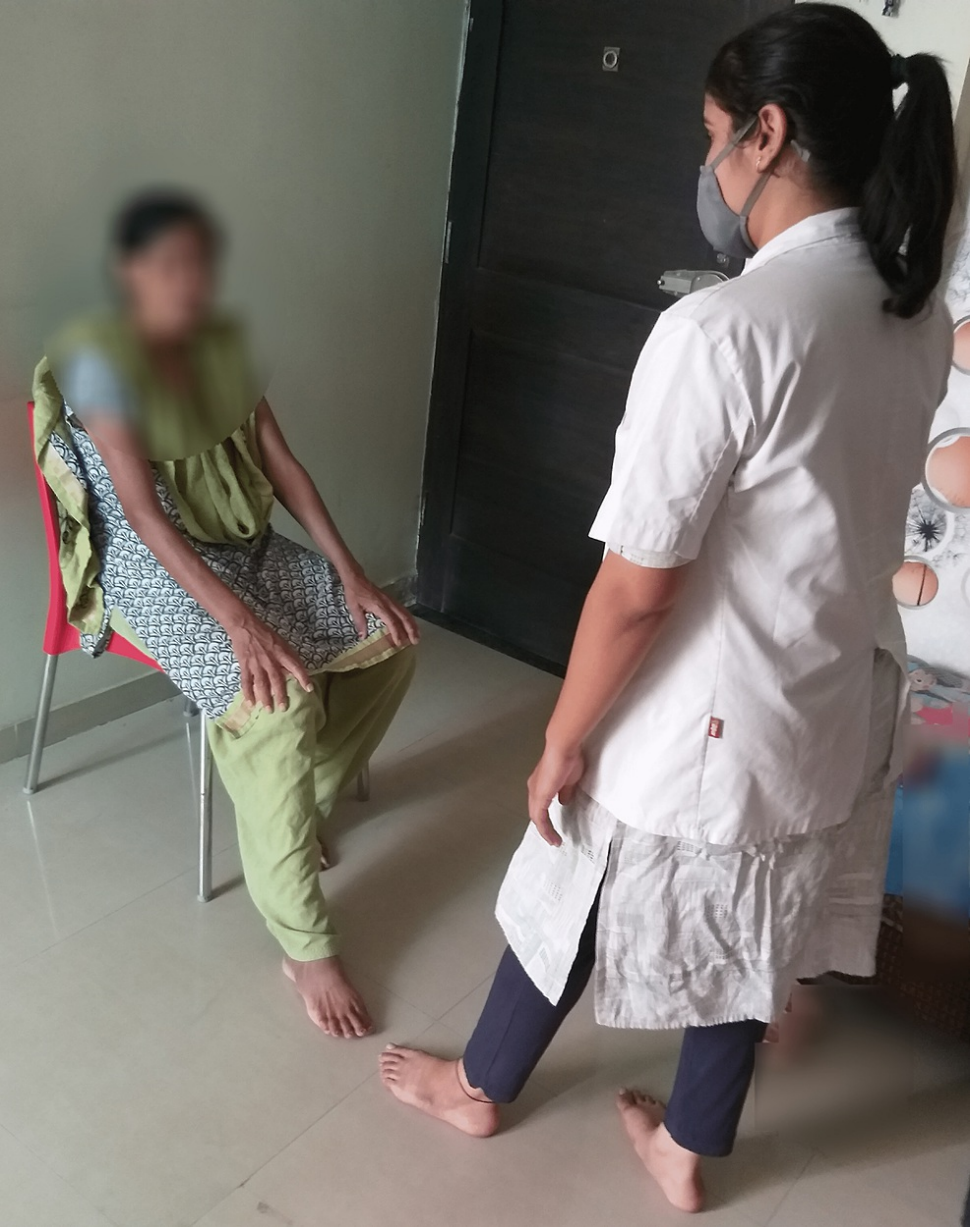
Sitting, dragging foot, and placing it on floor markers (Marked from 1 to 5) and with each repetition saying any sentences or words related to any topic given by the therapist.

The participants, while seated, performed actions like dragging the foot, and placing it on floor markers (marked from 1 to 5), and with each repetition, they said any sentence or words related to a topic given by the therapist., such as "family," "children," "weather," etc. (Figure 2).

**Figure 3:**
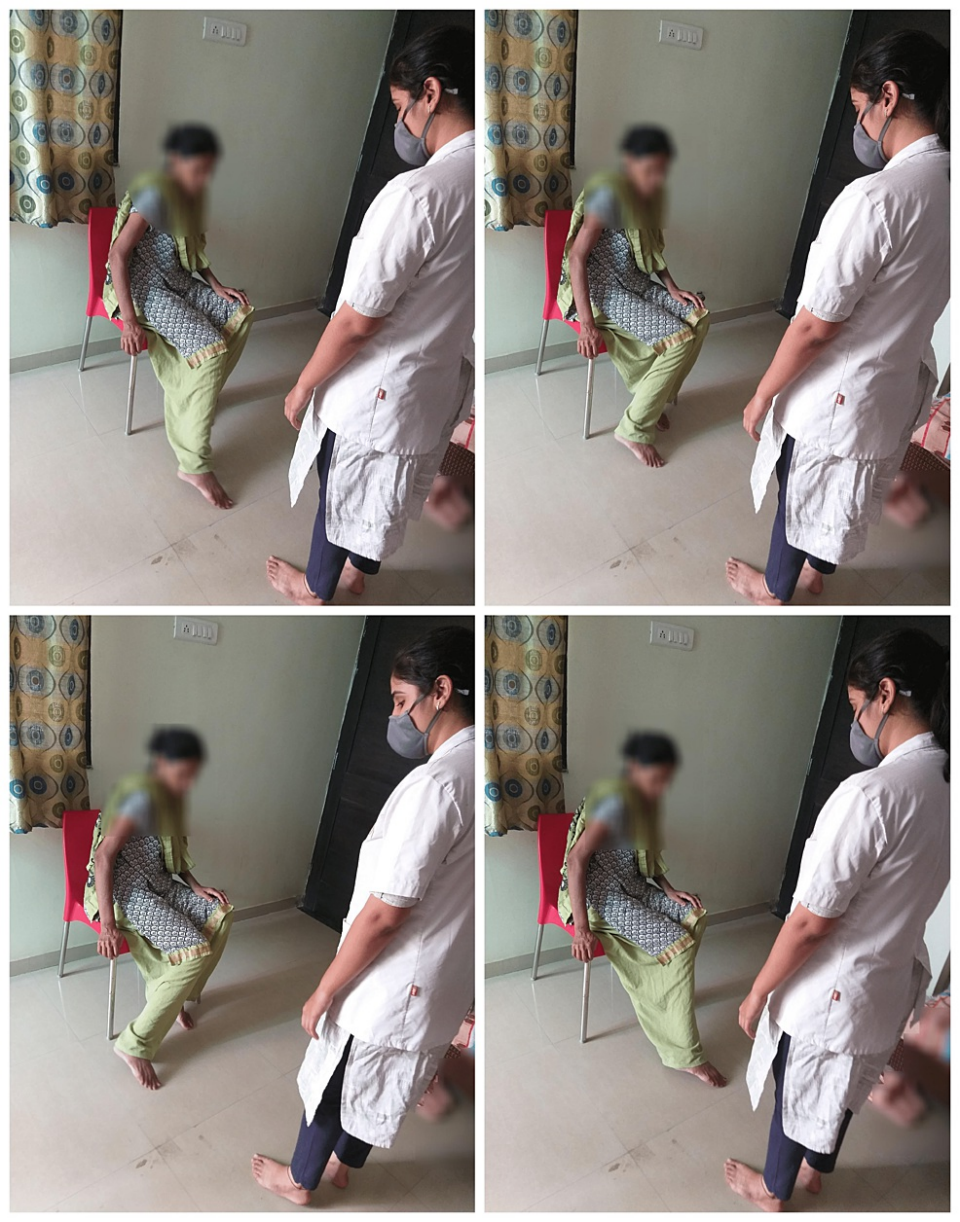
Sitting, drawimg any letter of the alphabet with foot (right/left) and name any word starting with the same letter.

The participants also performed actions like drawing any letter with their foot (right/left) while seated, and named any words starting with the same letter (Any 5) (Figure 3). 

**Figure 4:**
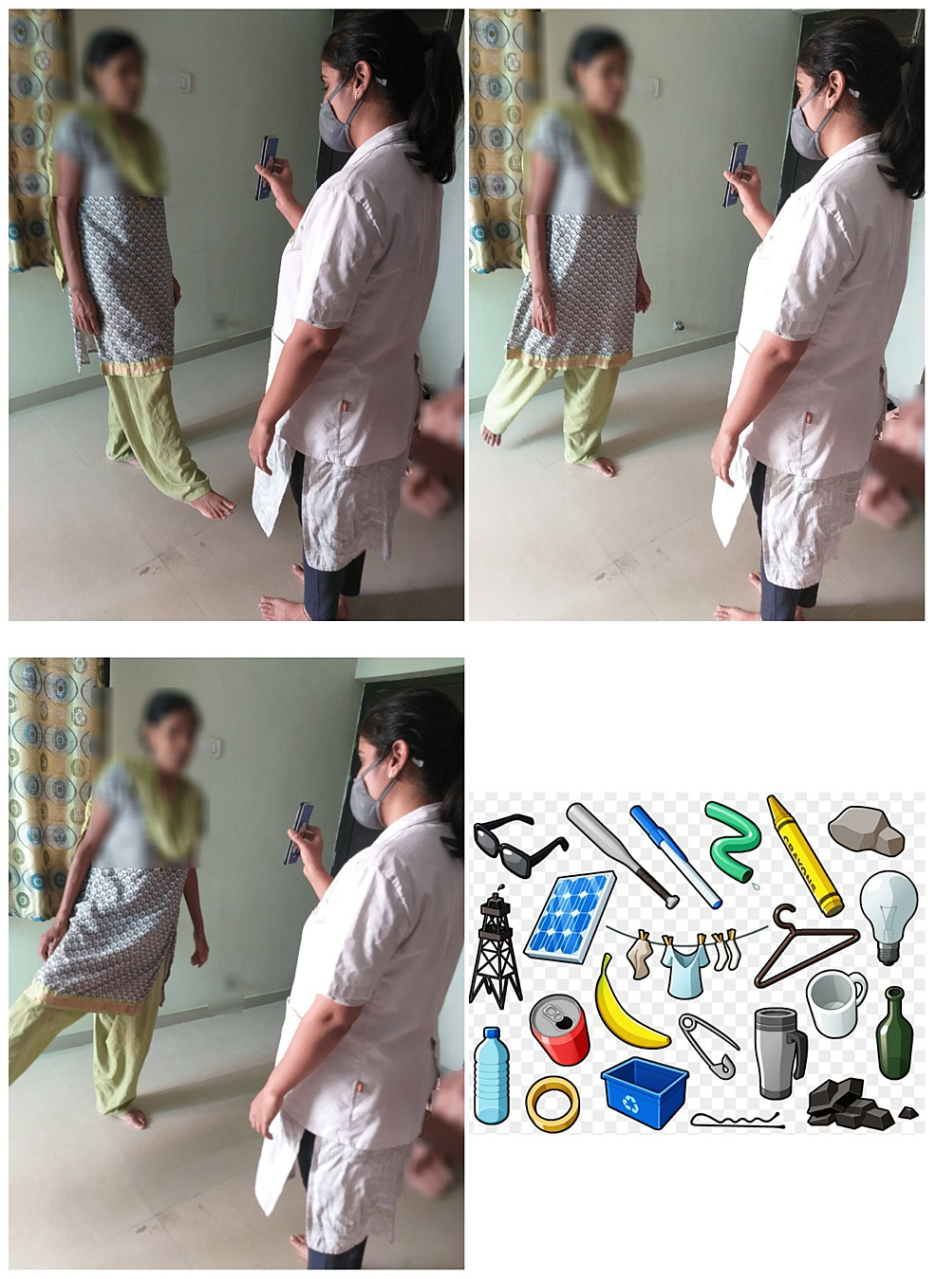
Standing, alternate hip flexion-extension, abduction-adduction, naming the objects shown in the picture.

While standing, with alternate hip flexion-extension, and abduction-adduction, the subjects named the objects shown in the picture as presented by the therapist (Figure 4).

**Figure 5:**
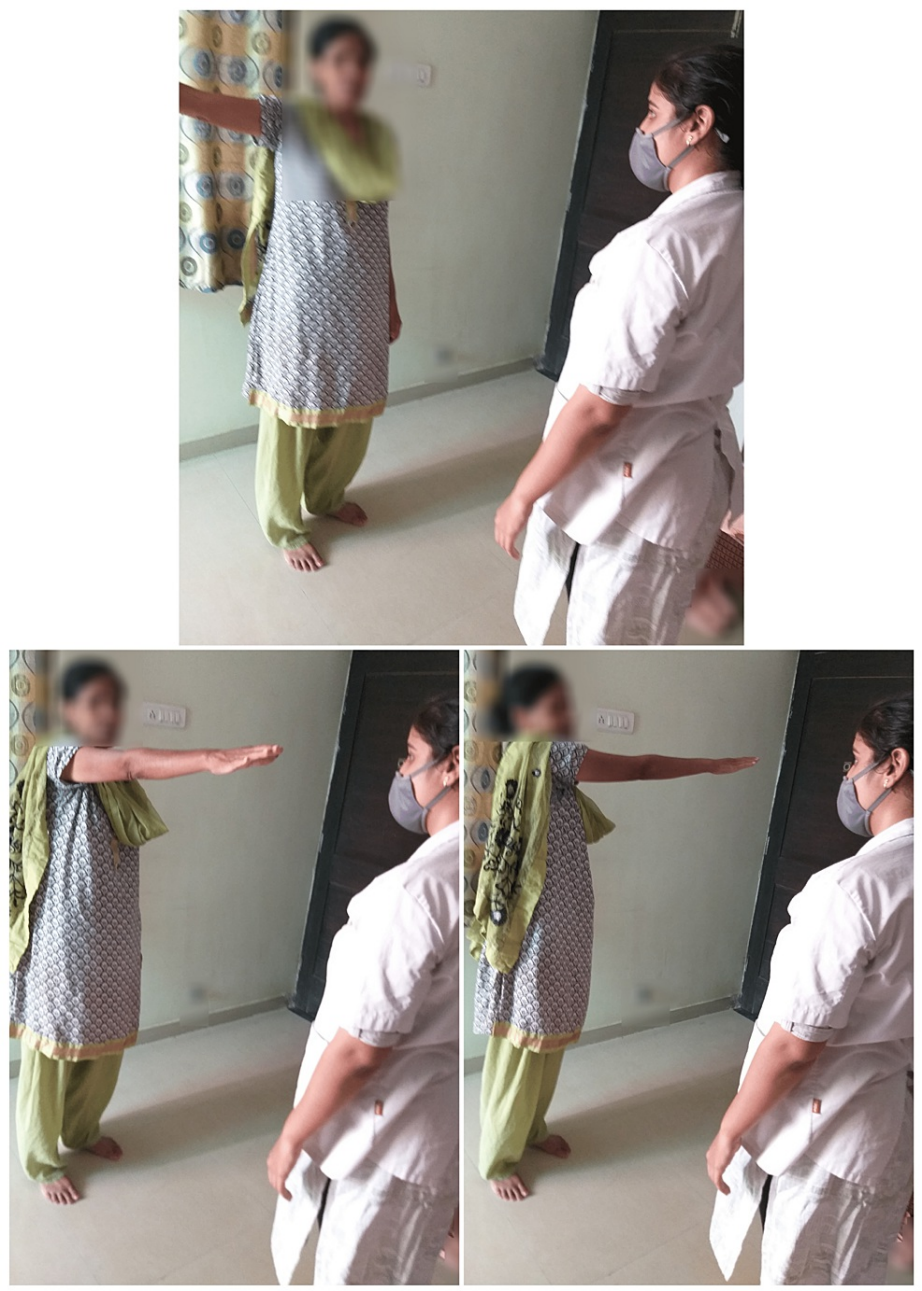
Standing, moving the arm in different directions, with each direction speaking out loudly days of the week.

Standing, subjects moved the arm in different directions, and with each direction, they named aloud the days of the week (Figure 5).

**Figure 6:**
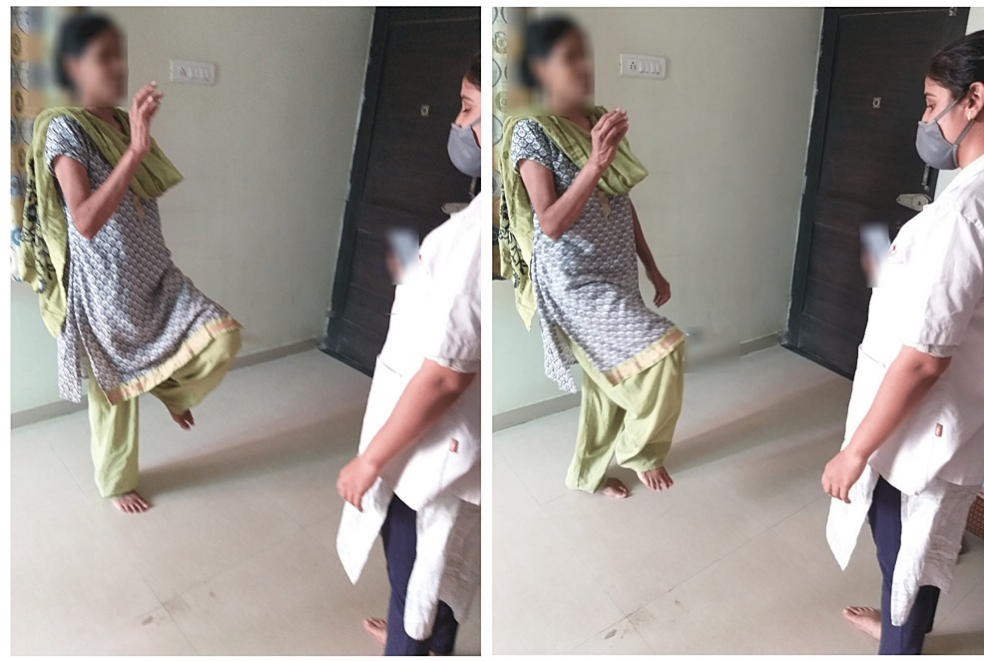
Spot marching, reciting multiplication tables of 11, 12, 13, 14 backwards in first, second, third, fourth weeks, respectively.

With spot marching, participants recited multiplication tables of 11,12,13,14 backward in the first, second, third, and fourth weeks, respectively (Figure 6).

**Figure 7:**
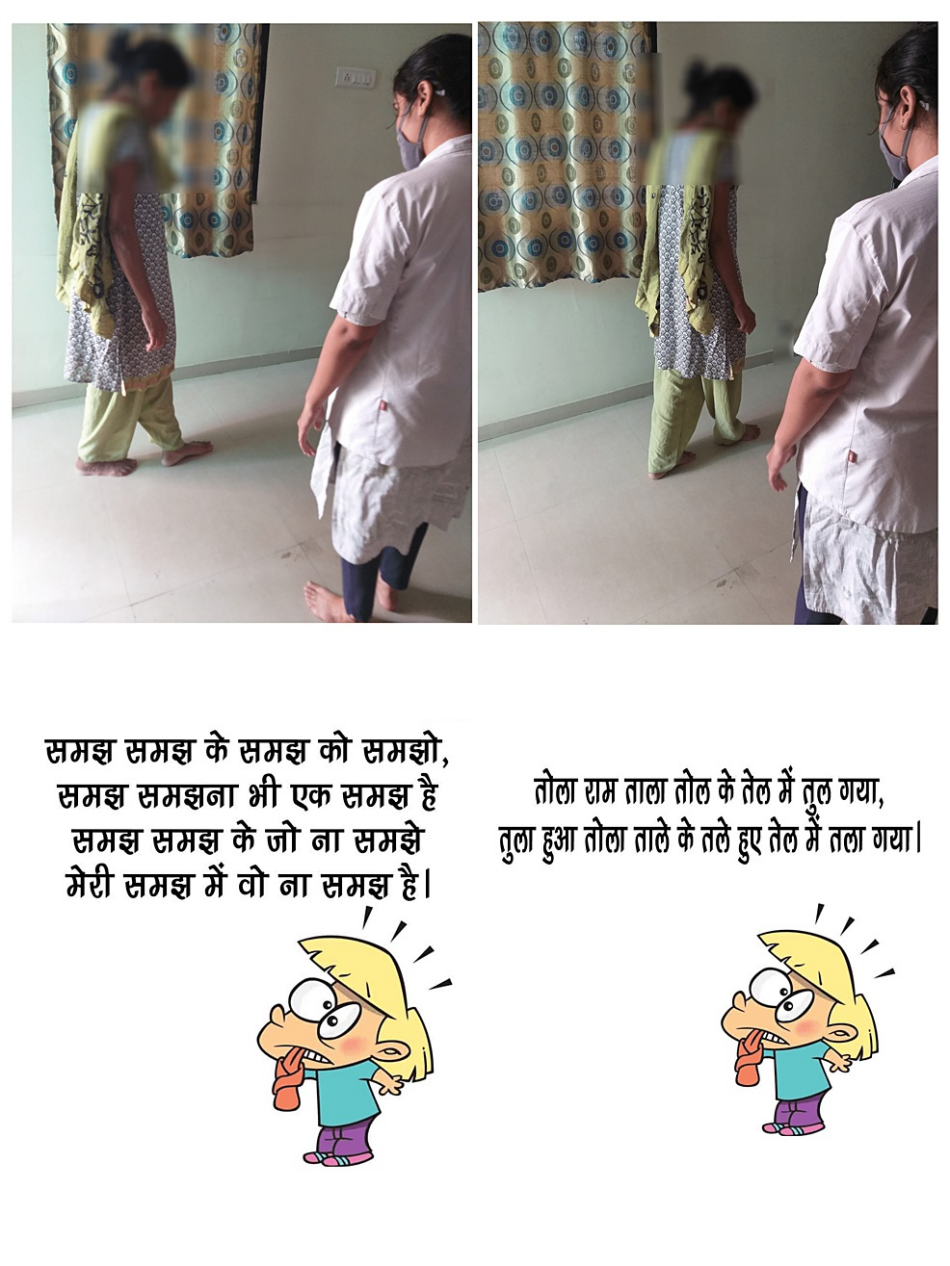
Tandem walking, reciting a tongue twister. English translation of Tongue Twister 1 (left side): understand the understanding by understanding it, to understand understanding is also an understanding. who doesn't understand understanding by understanding it according to my understanding, he has no understanding. English translation of Tongue Twister 2 (right side): Tola Ram weighed the lock and got weighed in the oil The weighed Tola got fried in the fried oil of the lock

Other activities done were: walking forward while listening to sounds in the surroundings such as fan, chirping of birds, rumbling of leaves, etc.; while walking by evading obstacles, the subjects were given any scenario and were told to think, analyze, and tell what will they do; while walking backward, the subjects had to draw a clock and were told to put in all the numbers and set the time to 4 past 15; While tandem walking, they recited a tongue twister (Figure 7).

The subjects performed a dance step and spoke aloud the names of the months in a year. While climbing stairs, a song was played, and then suddenly stopped. The subjects were asked to continue singing from that line. In another exercise, before ascending stairs, a specific sequence of tactile stimuli (different textures) was presented to the subjects. Later, after completing the descent, they were asked to identify and state the sequence of the textures presented. While ascending stairs backwards, subjects executed serial 7 subtractions, forward digit span, and backward digit span. The subjects also performed orientation exercises in a room with the help of a map. The subjects followed the instructions and described something that they could find in a convenience store, house, room, etc.

Group B: AT

**Figure 8:**
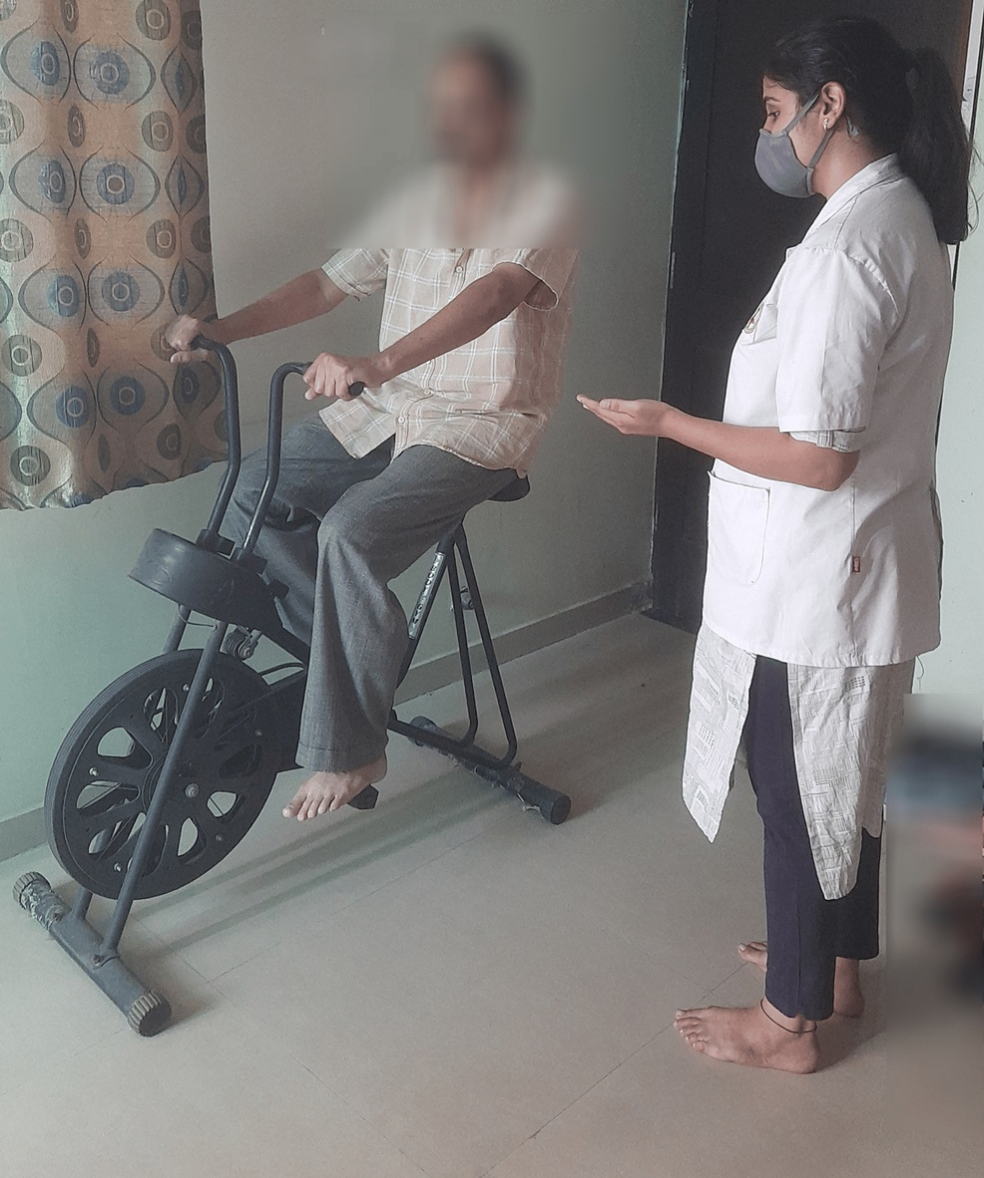
Subject riding a static cycle.

The training program was based on the WHO's 2010 Global Recommendations On Physical Activity For Health and the ACSM guidelines [19,28]. Literature is divided over the duration of AT required to obtain a positive outcome. One study reported a positive outcome within six weeks [29]. Participants underwent AT for 45 min, five days per week. The participant's maximal and resting heart rates determined the training intensity. The warm-up consisted of stretching exercises and walking for 5-10 minutes. The active phase involved the treadmill, bicycle, and walking exercises (Figure 8). Participants were encouraged to walk quickly in a single uninterrupted bout (45 minutes for five days); intensity and duration were initiated in a stepwise manner [13,19]. During the first week, the subjects walked for 30 minutes at 9-10 on the Borg Rating of Perceived Exertion Scale (BRPES) contemplated as mild intensity. In the second week, the duration was raised to 45 min, the intensity being similar to the previous one. Throughout the rest of the session (four weeks), they kept the 45-minute workout and raised the intensity of the exercise to a modest-vigorous effort, which corresponds to 12-14 on the BRPES. Participants were taught how to use the BRPES and keep track of their exercise intensity and frequency in a diary [19]. The cool-down period was of 10-minute duration.

Statistical analysis

The level of significance for the statistical analysis was set at p<0.05, and descriptive and inferential statistics were performed using the Chi-square test, student paired and unpaired t-tests, and the software used for the analysis was IBM SPSS Statistics for Windows, Version 27.0 (Released 2020; IBM Corp., Armonk, New York, United States) and GraphPad Prism 7.0 (GraphPad Software, San Diego, California, United States). The student t-test was used to compare groups A (DTT) and B (AT) to determine which training protocol was most efficient in improving cognitive function. Within groups A and B, a paired t-test was used to compare pre and post-scores. An unpaired t-test was used to compare the post-mean difference scores between groups A and B.

## Results

The distribution of subjects according to baseline characteristics is shown in Table 2. The mean age (P<0.17), sex (P<0.74), hand dominance (P<1.00), and education (P<0.95) of the two groups were very marginally different.

**Table 2:** Distribution of patients according to baseline characteristics. NS: non-significant

Baseline Characteristics	Group A	Group B	p-value
Age (years)	64.05±3.17	65.50±3.44	0.17, NS
Gender			
Male	13 (65%)	11(55%)	0.74, NS
Female	7 (35%)	9 (45%)	
Hand Dominance			
Right-handed	15 (75%)	16 (80%)	1.00, NS
Left-handed	5 (25%)	4 (20%)	
Education (years)	13.75±2.43	13.80±2.72	0.95, NS

**Figure 9:**
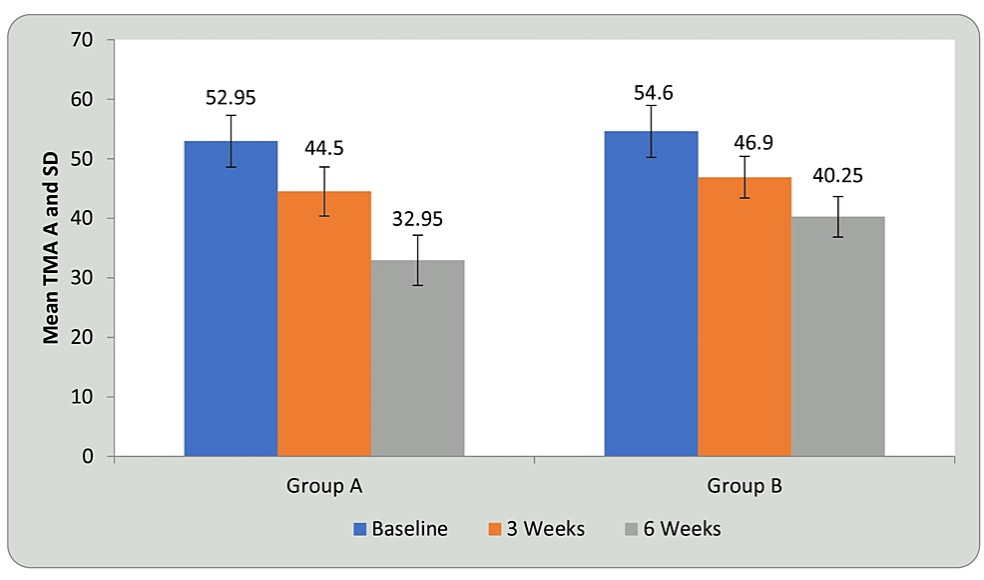
Comparison of TMT-A in group A and group B at three weeks and six weeks when compared with baseline TMT-A: trail making test A; SD: standard deviation

**Figure 10:**
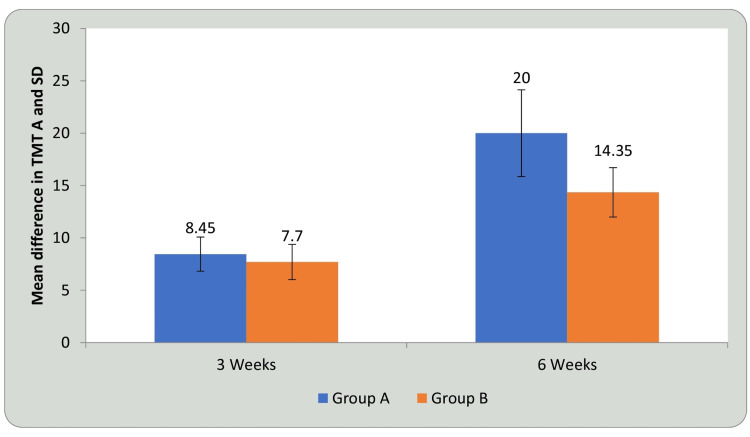
Comparison of mean difference in TMT-A in group A and group B at three weeks and six weeks. TMT A: trail making test A, SD: standard deviation

Tables 3, 4 and Figures 9, 10 show statistical evidence for the impact of treatment on TMT-A scores in groups A and B at three weeks and six weeks compared with baseline. Table 3 and Figure 9 depict a statistical analysis of the TMT-A scores and the significant value of comparison between the groups at baseline, third week, and sixth week using student’s paired t-test. In groups A and B, there was a considerable decrease in the duration required to complete the test, indicating improvement in CF following treatment compared to before treatment (P<0.0001). Table 4 and Figure 10 shows a comparison of the mean difference in TMT A scores in groups A and B at three weeks and six weeks using student’s unpaired t-test. When the mean difference in duration required to complete the test was compared between the two groups at three weeks and six weeks, it was observed that there was no significant difference at three weeks (P<0.16); however, the significant difference was noted at six weeks, i.e., following completion of the intervention (P<0.0001) and the DTT group performed better than the AT group (P<0.0001), showing a significant difference between both but DTT > AT

**Table 3:** Comparison of TMT-A in group A and group B at three weeks and six weeks when compared with baseline (Student’s paired t-test) TMT-A: trail making test A; S: significant

	Baseline	3 Weeks	6 Weeks
Group A	52.95±4.35	44.50±4.12	32.95±4.22
Mean Difference		8.45±1.63	20±4.14
t-value		23.07; P=0.0001, S	21.59; P=0.0001, S
Group B	54.60±4.36	46.90±3.50	40.25±3.40
Mean Difference		7.70±1.68	14.35±2.36
t-value		20.38; P=0.0001, S	27.10; P=0.0001, S

**Table 4:** Comparison of mean difference in TMT-A in group A and group B at three weeks and six weeks (Student's unpaired t-test) TMT-A: trail making test A; NS: non-significant; S: significant

Weeks	Group A	Group B	t-value
3 Weeks	8.45±1.63	7.70±1.68	1.42; P=0.16, NS
6 Weeks	20±4.14	14.35±2.36	5.29; P=0.0001, S

**Figure 11:**
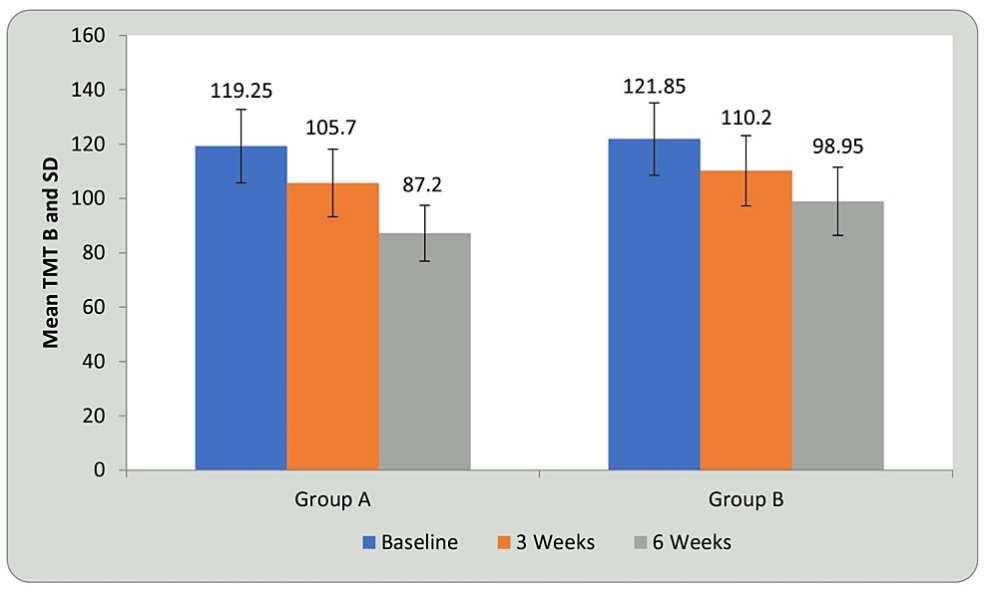
Comparison of TMT-B in group A and group B at three weeks and six weeks when compared with baseline. TMT-B: trail making test B; SD: standard deviation

**Figure 12:**
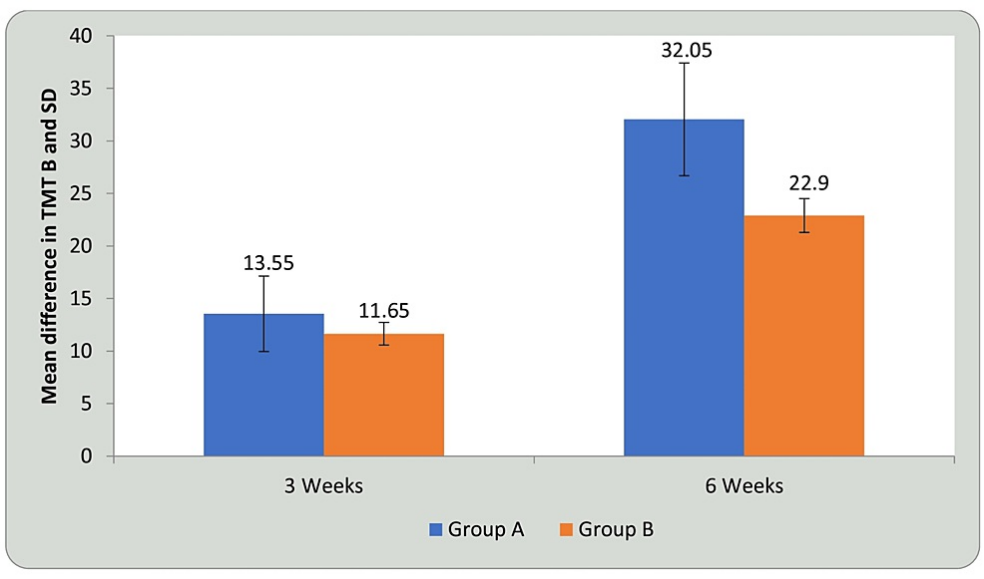
Comparison of mean difference in TMT-B in group A and group B at three weeks and six weeks TMT-B: trail making test B; SD: standard deviation

Tables 5, 6 and Figures 11, 12 show statistical evidence for the impact of treatment on TMT-B scores in groups A and B at three weeks and six weeks compared with baseline. Table 5 and Figure 11 depicts a statistical analysis of the TMT-B scores and the significant value of comparison between the groups at baseline, third week, and sixth week using student’s paired t-test. In groups A and B, there was a considerable decrease in the duration required to complete the test, indicating improvement in CF following treatment compared to before treatment (P<0.0001). Table 6 and Figure 12 shows a comparison of the mean difference in TMT B scores in groups A and B at three weeks and six weeks using student’s unpaired t-test. When the mean difference in duration required to complete the test was compared between the two groups at three weeks and six weeks, it was observed that there was a significant difference both at three weeks (P<0.029) and six weeks, i.e., following completion of the intervention (P<0.0001) and the DTT group performed better than the AT group (P<0.0001), showing a significant difference between both but DTT > AT. 

**Table 5:** Comparison of TMT B in group A and group B at three weeks and six weeks when compared with baseline (Student's paired t-test) TMT-B: trail making test B; S: significant

	Baseline	3 Weeks	6 Weeks
Group A	119.25±13.52	105.70±12.44	87.20±10.28
Mean Difference		13.55±3.59	32.05±5.36
t-value		16.87; P=0.0001, S	26.71; P=0.0001, S
Group B	121.85±13.35	110.20±12.91	98.95±12.53
Mean Difference		11.65±1.08	22.90±1.61
t-value		47.82; P=0.0001, S	63.25; P=0.0001, S

**Table 6:** Comparison of mean difference in TMT-B in group A and group B at three weeks and six weeks (Student's unpaired t-test) TMT-B: trail making test B; S: significant

Weeks	Group A	Group B	t-value
3 Weeks	13.55±3.59	11.65±1.08	2.26; P=0.029, S
6 Weeks	32.05±5.36	22.90±1.61	7.30; P=0.0001, S

**Figure 13:**
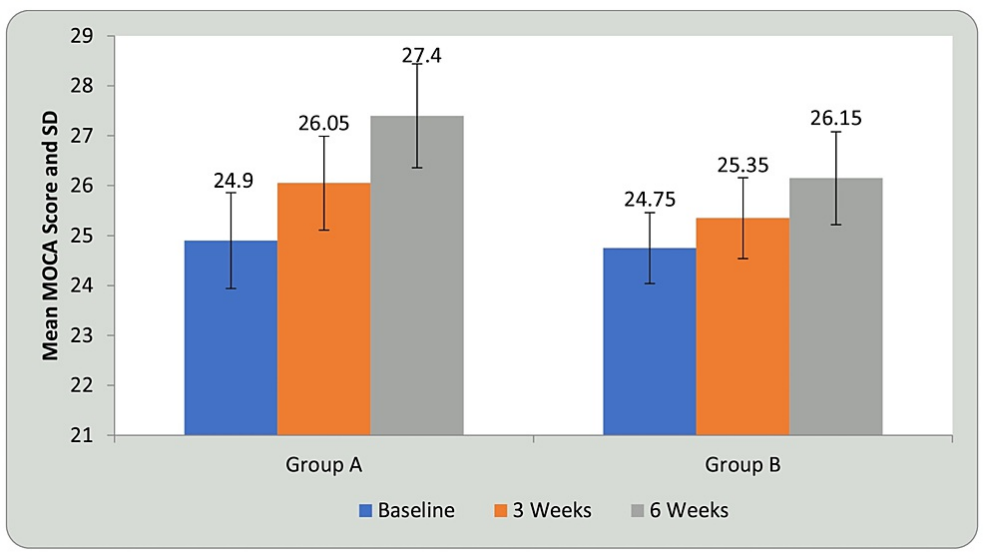
Comparison of MoCA in group A and group B at three weeks and six weeks when compared with baseline MoCA: Montreal Cognitive Assessment; SD: standard deviation

**Figure 14:**
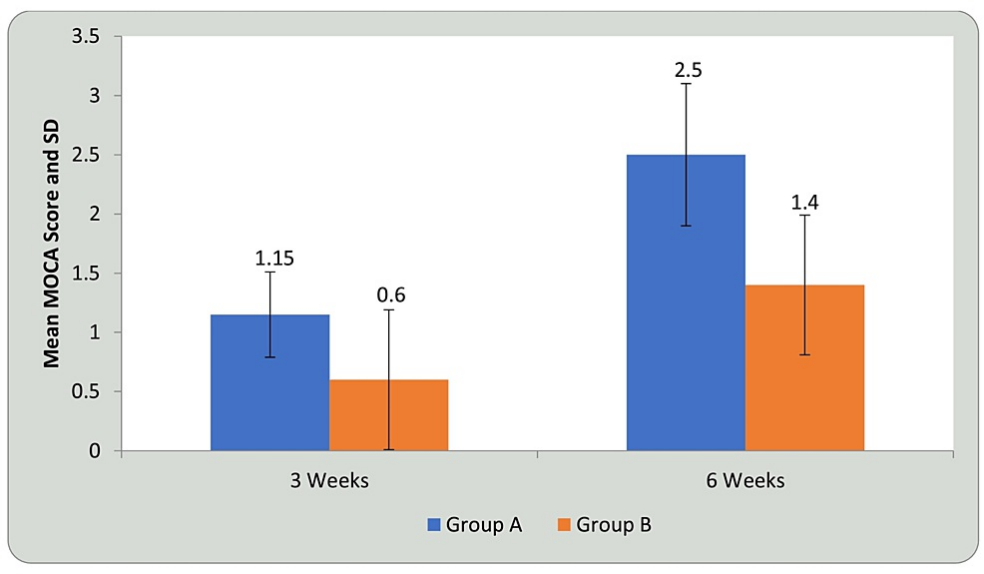
Comparison of mean difference in MoCA in group A and group B at three weeks and six weeks MoCA: Montreal Cognitive Assessment; SD: standard deviation

Tables 7, 8 and Figures 13, 14 show statistical evidence for the impact of treatment on MoCA scores in groups A and B at three weeks and six weeks compared with baseline. Table 7 and Figure 13 depict a statistical analysis of the MoCA scores and the significant value of comparison between the groups at baseline, third week, and sixth week using student’s paired t-test. In groups A and B, there was a considerable increase in the MoCA scores, indicating improvement in CF following treatment compared to before treatment (P<0.0001). Table 8 and Figure 14 depict the comparison of the mean difference in MoCA scores in groups A and B at three weeks and six weeks using student’s unpaired t-test. When the mean difference in MoCA scores was compared between the two groups at three weeks and six weeks, it was observed that there was a significant difference both at three weeks (P<0.001) and six weeks, i.e., following completion of the intervention (P<0.0001) and the DTT group performed better than the AT group (P<0.0001), showing a significant difference between both but DTT > AT.

**Table 7:** Comparison of MoCA in group A and group B at three weeks and six weeks when compared with baseline (Student's paired t-test) MoCA: Montreal Cognitive Assessment; S: significant

	Baseline	3 Weeks	6 Weeks
Group A	24.90±0.96	26.05±0.94	27.40±1.04
Mean Difference		1.15±0.36	2.50±0.60
t-value		14.03; P=0.0001, S	18.42; P=0.0001, S
Group B	24.75±0.71	25.35±0.81	26.15±0.93
Mean Difference		0.60±0.59	1.40±0.59
t-value		4.48; P=0.0001, S	10.46; P=0.0001, S

**Table 8:** Comparison of mean difference in MoCA in group A and group B at three weeks and six weeks (Student's unpaired t-test) MoCA: Montreal Cognitive Assessment; S: significant

Weeks	Group A	Group B	t-value
3 Weeks	1.15±0.36	0.60±0.59	3.50; P=0.001, S
6 Weeks	2.50±0.60	1.40±0.59	5.77; P=0.0001, S

## Discussion

Cognition encompasses a range of mental functions that enable us to perceive, process, and interact with our surroundings [11,30]. As a result, having healthy CF is critical for human existence. Processing speed and memory are two CFs proven to decline with aging. The number of people with poorer cognitive abilities is expected to rise in the coming years, mandating the establishment of comprehensive cognitive improvement interventions that aid in optimizing cognitive reserve and resistance to neurodegeneration [11]. The current study attempts to determine the inclusion of which training strategy, either DTT or AT, would be more effective in improving CF in a healthy elderly population. TMT-A, TMT-B, and MoCA were used as outcome measures to assess cognition.

DTT has been proven in numerous trials to be useful in improving cognition in the older population [5,13-15,19,22,31-34] and many studies have shown the effectiveness of AT on cognition in the elderly population [4,13,19,20,35-37]. There is a lack of enough evidence to show the importance of TMT in assessing CF. Very few studies have shown how DTT improves MoCA scores [38-40] and TMT scores [41,42], especially in the healthy elderly population. Similarly, very few studies have shown how AT improved MoCA scores [29,43,44] and TMT scores [19]. Few studies have been conducted to test the efficacy of DTT versus AT in improving CF in healthy elderly people, mostly outside India [45].

TMT-A is used for evaluating WM. The time required for completion of the test is recorded as the test result [24]. The mean duration needed to finish the test was reduced from 52.95, 44.50 to 32.95 for group A and from 54.60, 46.90 to 40.25 for group B at baseline, third week, and sixth week, respectively. However, by comparing the mean difference of TMT-A in the third and sixth week, mean values increased from 1.63 to 4.14 in Group A and 1.68 to 2.36, respectively, in Group B, and a significant difference was found in the two groups post six weeks of intervention. TMT-B is used for evaluating EF. The mean duration required for completion of the test was reduced from 119.25, 105.70 to 87.20 for group A and 121.85, 46.90 to 40.25 for group B at baseline, third week, and sixth week, respectively, after six weeks of training. By comparing the mean difference of TMT-B in the third and sixth week, mean values increased from 3.59 to 5.36 in Group A and 1.08 to 1.61, respectively, in Group B, and a significant difference was found in the two groups in the third and sixth week post intervention. These results reveal that both groups improved significantly after treatment, although group A improved the most.

MoCA is used to assess general CF. It examines cognitive processes such as memory, language, executive functions, visuospatial abilities, calculation, abstraction, attention, and concentration. It has a total score of 30, with higher numbers indicating better CF. A normal score is considered 26 or higher. MCI is characterized by a score of 19 to 25. MoCA is associated with higher sensitivity (90%) and specificity (87%) in the detection of cognitive decline or MCI [25]. The mean score increased from 24.90, 26.05 to 27.40 for group A and 24.75, 25.35 to 26.15 for group B at baseline, third week, and sixth week, respectively, after six weeks of training. By comparing the mean difference of MOCA in the 3rd and 6th week, mean values increased from 0.36 to 0.60 respectively in Group A. However, in Group B, the mean difference was the same in the third and sixth week, i.e., 0.59, and a significant difference was found in the two groups in the third and sixth week post intervention. Thus, significant improvement was seen post treatment in CF in both the groups, but more in group A. A study by Park showed that the MOCA scores changed from 22.53 ± 2.15 prior to intervention to 23.23 ± 2.24 post intervention in the DTT group after eight weeks of training [40].

The ability of a person to accomplish two activities simultaneously, such as cognitive and motor tasks, is referred to as DT [31,46]. Given the necessity of dealing with DT on a routine basis (as most routine tasks need the concurrent performance of the motor and cognitive tasks), as well as observed deterioration in performance as the individual ages, it appears evident that including the DT approach in training programs could be effective in increasing elderly folk's ability to walk and postural control while dealing with CF or addressing DT situations [32,47]. Even though divided attention diminishes with age, it is needed for several routine activities [15]. Compared with control groups, who are trained only for walking, DTT administered for six weeks improve memory in elderly individuals with a history of falls [33,48]. It is effective in improving general CF [17,49], EF [50,51], processing speed [26,52], visual, and verbal episodic memory, and sustained visual attention [5,26].

Aerobic activities such as cycling, walking, and running are included in treatment plans to help people with CF, particularly the elderly [11]. According to neuroimaging studies, AT increases volume in both gray and white matter over six months, notably in the prefrontal cortex (PFC), which is more sensitive to the impact of aging. In EF, the PFC is hypothesized to play a key role [36]. Aging affects the hippocampus and PFC, which can impair episodic memory, WM, attention encoding, and retrieval. As stated by Jardim et.al., “Daily physical activity improves brain structure and function, particularly in elderly people, with alterations occurring at the molecular/cellular, brain structure/function, mental states, and higher order behavioral levels, all of which contribute to the mitigation of functional losses" [5]. Di Liegro et.al. stated that “endurance activities such as long-duration aerobic activity (running) increase the circulating growth factors like insulin-like growth factor 1 (IGF-1) as well as neurotrophins like brain-derived neurotrophic factor (BDNF), both of which have an impact on the brain throughout development and in maturity" [35].

In older people, higher aerobic fitness levels were linked to the preservation of left and right hippocampal volume as well as better performance on a spatial memory test, demonstrating a three-part relationship between aerobic fitness, hippocampus volume, and memory functions [20,36]. According to meta-analyses, AET can improve a myriad of CFs in healthy elderly individuals, including executive skills, episodic memory, processing speed, and selective attention [13,22]. 

The study could be expanded in the future to include an equal number of males and females and compare the results of males and females, studying the efficacy of the two interventions in relation to one another while taking into account the different cognitive domains and running for a longer duration to determine the precise length of time required to improve CF using either technique. Limitations of the study were that only healthy people were included in it, the study was conducted on a small population, and unequal numbers of men and women were recruited.

A brief description of some of the studies including population, intervention, outcome measures, and findings is given in Table 9.

**Table 9:** Description of some studies including population, intervention, outcome measures, and findings EG: experimental group; CT: cognitive training; PT: physical training; CG: control group; TMT-A: trail making test A; TMT-B: trail making test B; CS: cognitive stimulation; AE: aerobic exercise; AET: aerobic exercise training; PAL: paired associate learning; RVP: rapid visual information processing; CERAD: consortium to establish a registry for Alzheimer’s disease; TUG: timed up and go test; 6MWT: six-minute walk test; 30 CST: 30-seconds chair stand test; DT: dual task; MCI: mild cognitive impairment; DTT: dual task training; ROM: range of motion; EFT: executive function training; EF: executive function; WMT: working memory training; WM: working memory; SDAEWMT: simultaneous dual aerobic exercise and working memory training; RAPM: Raven’s advanced progressive matrix; WMS-R: Wechsler memory scale-revised; FAB: frontal assessment battery; AD: Alzheimer’s disease; TG: training group; CF: cognitive function; MoCA: Montreal cognitive assessment; GDS: geriatric depression scale; CSR: chair sit and reach; STM: short term memory; CCT: computerized cognitive training; GNPT®: Gutmann Neuropersonal Trainer R; COMB: combined training; WAIS-III: Wechsler adult intelligence scale III; ROCF: Rey-osterrieth complex figure; RAVLT: Rey auditory verbal learning test; BNT: Boston naming test; MoCA 5-min: Montreal cognitive assessment five-minute; MMSE: mini-mental state exam; MT: motor tasks; STT: single task training; IG: intervention group; QoL: quality of life; HRQoL: health-related quality of life; PSQI: Pittsburgh sleep quality index; CAP: cognitive and physical training group; COG : cognitive training group; WAIS: Wechsler adult intelligence scale; EP: exercise program; MoCA-K: Montreal cognitive assessment- Korean version; K-CWST: Korean-color word Stroop test; K-GDS: Korean- geriatric depression scale; RT: resistance training; ST: single task; AT: aerobic training; DSST: digit symbol substitution task; WAIS-R: Wechsler adult intelligence scale-revised; ET: exercise training

Study	Population	Intervention	Outcome measures	Findings
Párraga-Montilla et al, 2021 [12]	43 older women with a mean age of 80.86 ± 5.03 years	60 minutes five times/week, for eight weeks EG 1 (n=10): CT EG 2 (n=10): PT+CT EG 3 (n=12): PT [Note: Protocol for PT session consisted of standard warm-up (10 minutes), active phase (40 minutes), and cool down (10 minutes) for EG2 and EG3.] CG (n=11): no training	Cognitive variables: Stroop test, D2 test, and TMT-A and TMT-B. physical variables: handgrip strength, two-minute step test, and visual–acoustic reaction time	It was evident that eight weeks of PT, CT, or combined PT+CT, boosted the physical and cognitive capacities of the three EGs whereas these capacities were reduced in CG.
Jardim et al., 2021 [5]	72 community-dwelling older adults with age more than 59 years)	24 group sessions, 75 minutes, two times/week for 12 weeks. Dual-task exercise (DTEx) (n=41): PT+CS. PT involved warm-up (10 minutes), AET (30 minutes), resistance exercise (30 minutes), and stretching (5 minutes). CS involved multisensory stimulation with tasks that mainly included functional responses to sensory stimuli, verbal and visual memory, motor learning, speech, attention, inhibition, and semantic and phonological fluency CG (n=31): no training	cognitive assessment: PAL, RVP, CERAD word list memory. physical activity assessment: international physical activity questionnaire (IPAQ) version 8 functional exercise capacity assessment: TUG, 6MWT, 30 CST, walking while talking test (DT agility) quality of life (QoL) assessment: 36-item short-form health questionnaire (SF 36)	The findings showed that a DT combination of PT and CS can be effectively implemented to slow down the progression of age-related cognitive decline and enhance physical fitness and QoL in healthy elderly people.
Jin Hyuck Park, 2021 [42]	36 older adults with MCI with a mean age of 74.00 ± 6.00 years	16 sessions, 40 minutes, two times/week for eight weeks. EG (n=18): DTT (PT+CT). PT consisted of AET (ROM exercise for shoulder, elbow, wrist, knee, and ankle) and strengthening exercise (thera-band with low-intensity training and passing/throwing a ball). CT involved verbal fluency, attention, memory, a game of rock-paper-scissors, and calculation tasks. CG (n=18): CT which involved EFT such as planning a vacation, logical reasoning, calculation, and shopping for 10 minutes each.	TMT-B	The findings of the study suggests that DTT is more effective in improving EFs for older adults with MCI than the single-CT.
Takeuchi et al., 2020 [13]	93 older adults with a mean age of 65.9 ± 13:7 years old	60 minutes, four times/week, for 12 weeks WMT (n=30): the game suite “Brain Age: Concentration Training” by Nintendo (seven WM tasks were used). AET (n=33): AT using recumbent ergocycle, which included warm-up (five minutes), AET (45 minutes), and cool down (10 minutes). SDAEWMT (n=30): WMT+AET	RAPM (nonverbal reasoning), digit span (verbal WM), WMS-R (logical memory), digit cancellation task (attention), FAB (frontal lobe and EF), symbol search and digit symbol tasks (processing speed)	SDAEWMT improved EF in the frontal lobe, induced neural tissue changes in the brain areas controlling EF and increased WM-related brain activity in areas involved in attentional reorienting.
Parvin et al., 2020 [53]	26 AD patients with a mean age of 67.4 ± 8.8 years	24 workouts, 40–60 minutes, two times/week for 12 weeks TG (n=13): combined protocol, including simple brain activities (eyes-closed training and cognitive activities) and physical activities (muscle endurance, balance, and aerobic capacity) including warm-up (10 minutes), main exercises (20–40 minutes), and cool down (10 minutes). CG (n=13): no training	CF: MoCA, depression: GDS, strength: knee extensions, biceps curl, handgrip (in kg), functional ability: 30 CST (N=number of stands), TUG (seconds), flexibility: CSR (centimetre), aerobic fitness: 6-MWT (metre)	Significant improvements were noted in CF, particularly in STM and WM, attention, and EF, depression status, strength aerobic fitness, flexibility, and functional ability in TG compared to CG.
Castells-Sánchez et al., 2020 [19]	healthy physically inactive older adults with a mean age of 58.38 ± 5.47 years	n=82 45 minutes, five times/week for 12 weeks AET (n=25): walking CCT (n=23): home-based multidomain CCT using GNPT^®^, Spain COMB (n=19): AET+CCT for 90 minutes, five times/week for 12 weeks CG (n=15): no training	Stroop test, WAIS-III, TMT A and B, verbal fluency tests, ROCF, RAVLT, BNT, MoCA 5-min, MMSE	AET group showed improvements in WM and attention including the attention-speed domain, COMB improved attention, speed, and the attention-speed domain compared to CG. However, the CTT group did not show any cognitive change compared to CG.
Taşvuran Horata et al., 2020 [27]	32 community-dwelling older individuals 60-75 years old	60 minutes, two times/week for six weeks DTT (n=16): MT+CT STT (n=16): MT only MT: standing, single leg standing, walking (forward, sideways, backward), and reaching (forward, sideways) CT: recalling a sequence of numbers previously given, drawing a letter or a word on the floor by a foot, saying the previous number and next number from a number between 0 and 100 provided by the trainer, collecting numbers, counting forward and backward from any number between 0–100	CF: standardized mini-mental state exam (SMMSE), stroop test gait parameters: 10-meter walk test, TUG	DTT group exhibited remarkable differences in gait variables as well as CF, whereas STT showed improvements in gait variables only. These implies that DTT is a beneficial and efficient method for promoting normal gait and CF in older people.
Song and Yu, 2019 [43]	120 community-dwelling older individuals with MCI with a mean age of 75.78 ± 6.28 years	IG (n=60): moderate intensity AE 60 minutes, three times/week for 16 weeks which included warm-up (10 min; walking and stationary stretching exercises for trunk and limb joints at the upper and lower bodies), moderate-intensity stepping exercises (20–40 minutes; stepping up and down on a 10- cm-high stable stepping bench), and cool down (10 minutes; walking and stationary stretching exercises). CG (n=60): health education program 45 minutes, two times/week for 16 weeks	MoCA (CF), QoL-AD (HRQoL), GDS (depression), PSQI (sleep quality)	IG demonstrated remarkably greater improvement in CF and HRQOL than the CG when compared pre and post-intervention. The exercise–cognition relationship was significantly mediated by reduced depressive symptoms and improved sleep quality.
Joubert and Chainay, 2019 [16]	48 healthy older adults with a mean age of 69.58 ± 3.36 years	60 minutes, two times/week for eight weeks CAP (n=16): CT+PT COG (n=16): CT only CG (n=16): no training CT: EFT (three games: basketball ball, tower of Hanoi, and long live alternation) and WMT (four games: birdsongs, subsets, waiter please, and game of heraldry) PT: walking on a treadmill for one hour	MoCA (general CF), RAVLT (memory), with TMT-A and TMT-B (switching), digit span subtest of the WAIS (verbal fluency, STM and WM), Victoria stroop test (visual inhibition), instrumental activities of daily living [IADL] (autonomy), McNair (memory disorders), GDS (depression), PSQI (sleep quality), 12-item short-form health questionnaire (SF 12) (QoL)	Results showed that the improvement in CF obtained by CAP and COG groups was sustained on a long-term basis, however, the improvement was more in the COG group as compared to the CAP group. This implies that CT and PT complement each other in terms of short-term outcomes, but when it comes to long-term outcomes CT is more advantageous.
Amjad et al., 2019 [29]	40 MCI patients 50 years and above	60 minutes, three times/week for six weeks AET (n=21): included warm-up (5-10 minutes), AET using a stationary bicycle (20–40 minutes), and cool down (5-10 minutes) CG (n=19): gentle movements and general body stretching were advised to perform at home	Electroencephalogram (EEG) and neurocognitive assessments (MMSE, MoCA, TMT-A, and TMT-B)	AET group showed remarkable improvements in slowness and complexity of the EEG and in MMSE, MoCA, TMT A, and TMT B scores following completion of training, compared to CG.
Park, 2017 [40]	21 older adults with MCI 60 years and above	60 minutes, two times/week for eight weeks DTT (n=11): EP+CT simultaneously STT (n=10): EP+CT by turns per session EP: AE (drawing of the wrist, elbow, shoulder, ankle, knee, and walking motion) and strength exercise (pull the thera-band by hand, pull and push TheraBand by leg, pass the ball to the side and back and forth, and throw the ball). CT: count the number (1-100, sequentially and randomly), naming of pictures (flowers, fruits, and vegetables), calculation (addition, ones place, and tens place, and subtraction ones place and tens place), naming backward, and find the common of each picture (flowers, fruits, animals and vegetables).	MoCA-K (general CF), FAB and K-CWST (frontal lobe functioning), digit span subtest of the WAIS (attention and WM), and K-GDS (depression)	The DTT group showed significant improvements in general CF, frontal lobe functioning, attention, and WM as well as the reduction in depression compared to the STT group.
Coetsee and Terblanche, 2017 [54]	67 inactive older adults with a mean age of 62.7 ± 5.7 years	Three times/week for 16 weeks RT (n=22): upper and lower body resistance exercises using machines and free weights for 30 minutes. high-intensity aerobic interval training (HIIT) (n=13): four intervals of four-minute treadmill walking at 90–95% maximal heart rate (HRmax), interspersed by three-minute active recovery periods at 70% HRmax for 30 minutes. moderate continuous aerobic training (MCT) (n=13): continuous walking on a treadmill at 70–75% of HRmax for 47 minutes. CG (n=19): no training	CF: Stroop task physical function (PF): TUG and submaximal Bruce treadmill tests	MCT and RT proved to be superior to HIIT for the enhancement of EF in the elderly; whereas HIIT was most beneficial for the improvement in information processing speed.
Yokoyama et al., 2015 [41]	25 community-dwelling healthy, inactive older individuals 65 years and above	60 minutes, three times/week for 12 weeks DT (n=12): simultaneous MT+CT ST (n=13): simple RT and AT (MT only) MT: 15-minute mental gymnastics (complicated motion of the fingers), 25-minute RT, 10 minutes of AET, and 10-minute systemic flexibility exercise. CT: arithmetic tasks (serial subtraction), and Shiritori (Japanese word chain game)	Modified mini-mental state examination, TMT A	DT group showed substantial improvements in cognitive skills viz. "registration & recall," "attention," "verbal fluency & understanding," and "visuospatial skills" compared to the ST group. However, TMT scores were indifferent for both groups.
Eggenberger et al., 2015 [51]	71 older adults 70 years and above	60 minutes, two times/week for 24 weeks DANCE (n=24): virtual reality video game dancing MEMORY (n=22): treadmill walking with a simultaneous verbal memory exercise PHYS (n=25): treadmill walking	TMT-B (EF), executive control task (WM), PAL (long-term visual memory), logical memory subtest (story recall), and digit forward and backward from WMS-R for (long-term and short-term verbal memory), age concentration tests A and B (attention), TMT-A and, DSST from WAIS-R (information processing speed)	Concurrent CT and PT led to improvements in EF, especially "shifting attention" and "WM". All groups preserved their improvements in EF, episodic memory, and processing speed at the follow-up. This implied that EF improve more from simultaneous CT and PT than from PT alone.
Gill et al., 2014 [45]	39 older adults with a mean age of 72.8 ± 7.1 years	75 min,utes two times/week for 24 weeks dual-task aerobic training (DAE): AE+CT AE: AE only	TMT-A and TMT-B, DSST, auditory verbal learning test (AVLT)	After three months, AVLT total learning, and delayed recall, improved within both groups. Additionally, immediate recall improved in the DAE group and information processing speed in the AE group.
Nagamatsu et al., 2013 [21]	86 elderly women with subjective memory complaints with a mean age of 74.9 ± 3.5 years	60 minutes, two times/week for 24 weeks RT (n=28): biceps curls, triceps extension, seated row, latissimus dorsi pull downs, leg press, hamstring curls, and calf raises using Keiser pressurized air system. Others included minisquats, minilunges, and lunge walks AT (n=30): outdoor walking CG (n=28): stretching, ROM, and balance exercises, and functional sand relaxation techniques	RAVLT (verbal memory and learning), and computerized task for spatial memory	AET group showed significant improvements in verbal memory and learning compared to CG after six months of training. Both RT and AT groups showed improved spatial memory performance compared to CG. A significant correlation was found between spatial memory performance and overall physical capacity after intervention in the AET group.
Shatil et al., 2013 [49]	122 healthy older adults with a mean age of 76.83 ± 5.51 years	CT (n=33): CT with CogniFit program 40 minutes, three times/week for 16 weeks PT (n=31): aerobic warm-up (10 minutes), cardiovascular workout seated and standing (15 minutes), cool-down (five minutes), strength training (10 minutes), and flexibility training (five minutes) Combined training (n=29): CT+PT CG (n=29): book reading	CogniFit neuropsychological evaluation	Those who underwent CT (CT and combined training groups) exhibited considerable improvements in hand-eye coordination, WM and long-term memory, information processing speed, visual scanning, and naming compared to PT and control groups.
Theill et al., 2013 [50]	63 healthy older adults with a mean age of 71.8 ± 4.9 years	20 training sessions of 30 minutes each for 10 weeks simultaneous training (n=21): concurrent PT (walking on treadmill) + CT (verbal WMT) single WMT (n=16): only WMT which included computerized n-back training and serial position training, CG (n=26): no training	Continuous performance task (selective attention), PAL, an executive control task, standard progressive matrices (SPM) test (reasoning), operation span test (memory span), a digit-letter task (information processing speed)	The simultaneous training group showed improvements in cognitive performance in the trained WM task as well as in the executive control task, PAL task, and motor-cognitive DT, whereas the single WMT group showed increased performance only in the trained WM task and executive control task.
Nouchi et al., 2013 [22]	64 healthy older adults 60 years or older)	Three times/week for four weeks Combination ET (n=32): AET, strength training, and stretching) CG (n=32): no training	EF- Stroop test, verbal fluency task. episodic memory-logical memory, first and second names. WM- digit span forward and backwards. reading ability- Japanese reading test (JRAT). attention-digit cancellation task. processing speed- digit symbol coding, symbol search.	Combination ET groups showed improvements in EF, episodic memory, and processing speed compared to CG.

## Conclusions

Cognitive decline is common in elderly people due to the effects of aging on the brain. However, age-related decline in cognition could be prevented. DTT and AET are two of those interventions that help prevent cognitive decline. In this study, we compared the two training techniques to determine which one is the most effective. The effects of DTT and AET on cognitive functions were compared and it was observed that after six weeks of training, both groups showed significant improvement on all mentioned outcome measures. But Group A, which received DTT, showed statistically more improvement compared to Group B, which received AET. This study concludes that both types of interventions can be employed to improve cognition in the healthy elderly. However, DTT is more effective than AET in improving cognitive function.
